# A Gifsy prophage-encoded protein confers broad phage resistance in *Salmonella enterica* and is widely distributed across *Enterobacteriaceae*

**DOI:** 10.1128/aem.01384-25

**Published:** 2025-11-10

**Authors:** Jaap S. Bosma, Vincent Noireaux, Steven D. Bowden

**Affiliations:** 1 Department of Food Science and Nutrition, University of Minnesota 311837 , Saint Paul, Minnesota, USA; 2 School of Physics and Astronomy, University of Minnesota 5635, Minneapolis, Minnesota, USA; Universidad de los Andes, Bogotá, Colombia

**Keywords:** bacteriophage, *Salmonella*, phage resistance, Gifsy prophages, phage depolymerase, food safety

## Abstract

Phage Felix O1 (FO1) is one of the most widely used bacteriophages for targeting *Salmonella enterica* and is applied in both research and food safety settings. Despite its broad host range, we observed significantly reduced efficiency of plating (EOP) on a subset of *Salmonella enterica* strains, including serovar Typhimurium 4/74 (*S*. 4/74). We found that O-antigen–mediated steric hindrance contributes to this resistance, which can be partially overcome by supplementation with the SP6 phage tailspike protein (TSP). However, full restoration of the EOP required deletion of the Gifsy-1 prophage, leading to the identification of a single prophage-encoded defense gene, *gipd474* (Gifsy-encoded phage defense protein from strain *S*. 4/74). Heterologous expression of this gene in *Escherichia coli* increased resistance to multiple tailed phages. Bioinformatic analysis revealed that this gene is widely spread across the *Enterobacteriaceae*, suggesting a conserved role in phage resistance. Together, these findings uncover a dual barrier to FO1 infectivity in *S*. 4/74 and highlight *gipd474* as a novel, prophage-encoded phage defense gene.

**IMPORTANCE:**

Phage therapy and biocontrol are increasingly explored as alternatives to antibiotics, particularly against drug-resistant pathogens such as *Salmonella*. FO1 is a widely used lytic phage with broad activity across *Salmonella* serotypes. However, variability in phage infectivity limits its reliability against certain strains. Here, we report that *S*. 4/74 restricts FO1 through a combination of O-antigen–associated interference and a novel prophage-encoded resistance gene. This gene also impairs the infectivity of other tailed phages and is conserved across multiple *Enterobacteriaceae* genera. Our findings highlight the importance of prophage-encoded defense systems in shaping phage susceptibility and highlight the need to account for such elements when developing phage-based applications.

## INTRODUCTION

Antimicrobial resistance (AMR) is a major global health concern, responsible for causing 2.8 million antibiotics-resistant infections and more than 35,000 deaths annually in the United States alone (1). Among the most concerning contributors are multidrug-resistant (MDR) members of the *Enterobacteriaceae* family, including *Klebsiella pneumoniae*, *Escherichia coli*, *Salmonella* spp., *and Shigella* spp. (2). While *E. coli* and *K. pneumoniae* are the main contributors, drug-resistant nontyphoidal *Salmonella* (NTS) is emerging as a serious threat (3–5). With the decline of newly discovered antibiotics, bacteriophages have gained renewed interest as targeted antimicrobials (6–8). Phages are now applied more broadly, including within phage therapy, food safety, agriculture, and animal health (9–11).

The extensive application of phages has coincided with the discovery of a vast and diverse amount of prokaryotic immune systems, raising concerns about phage resistance (12–15). Many anti-phage defense systems are harbored within prophages, which are temperate phages integrated into the bacterial genome. Prophages have been shown to enhance host fitness, mediate horizontal gene transfer (HGT), support metabolic functions, influence ecological competition, and shape bacterial virulence (16–21).

In *Salmonella,* the Gifsy prophages (Gifsy-1, Gifsy-2, and Gifsy-3) are widespread, particularly among *Salmonella enterica* serovar Typhimurium (22, 23). However, prophage Gifsy-1 has also been found in other *S. enterica* lineages (24, 25). These prophages exhibit a highly mosaic structure resulting in diverse immunity regions, likely originating from intrachromosomal recombination/conversion events (26).

One of the most widely used phages in research and industry is *Salmonella* phage Felix O1 (FO1), which is commercially available in the biocontrol agent, PhageGuard (27). FO1 exhibits an extraordinarily broad host range, capable of infecting a wide range of *Salmonella* serotypes (28, 29). For phage infection to occur, FO1 must first recognize and bind to the host receptor. The somatic receptor for FO1 is the lipopolysaccharide (LPS); more specifically, the N-acetylglucosamine branch of the LPS outer core was found to be essential for phage–host interaction (30–32). In smooth *Salmonella* strains, long O-antigen chains can shield this receptor, reducing FO1 adsorption (30). This physical barrier can be overcome by tailspike protein (TSP), such as endo-rhamnosidase encoded by phage P22, which cleaves the O-antigen to enhance phage infectivity (33). A P22 TSP homolog was found in *Salmonella* phage SP6 and is predicted to function analogously (34, 35).

Despite the broad lytic activity of FO1, we observed that its efficiency of plating (EOP) was significantly reduced on a subset of *Salmonella* strains, suggesting the presence of infection barriers. This led us to further investigate these different *Salmonella* barriers that may contribute to FO1 resistance.

Here, we identify a previously uncharacterized prophage-encoded gene in *Salmonella* that confers resistance to multiple tailed phages, including FO1, and explore its distribution, function, and potential mechanism of action.

## RESULTS

### Cell-free expression and purification of SP6 TSP

**Fig 1:**
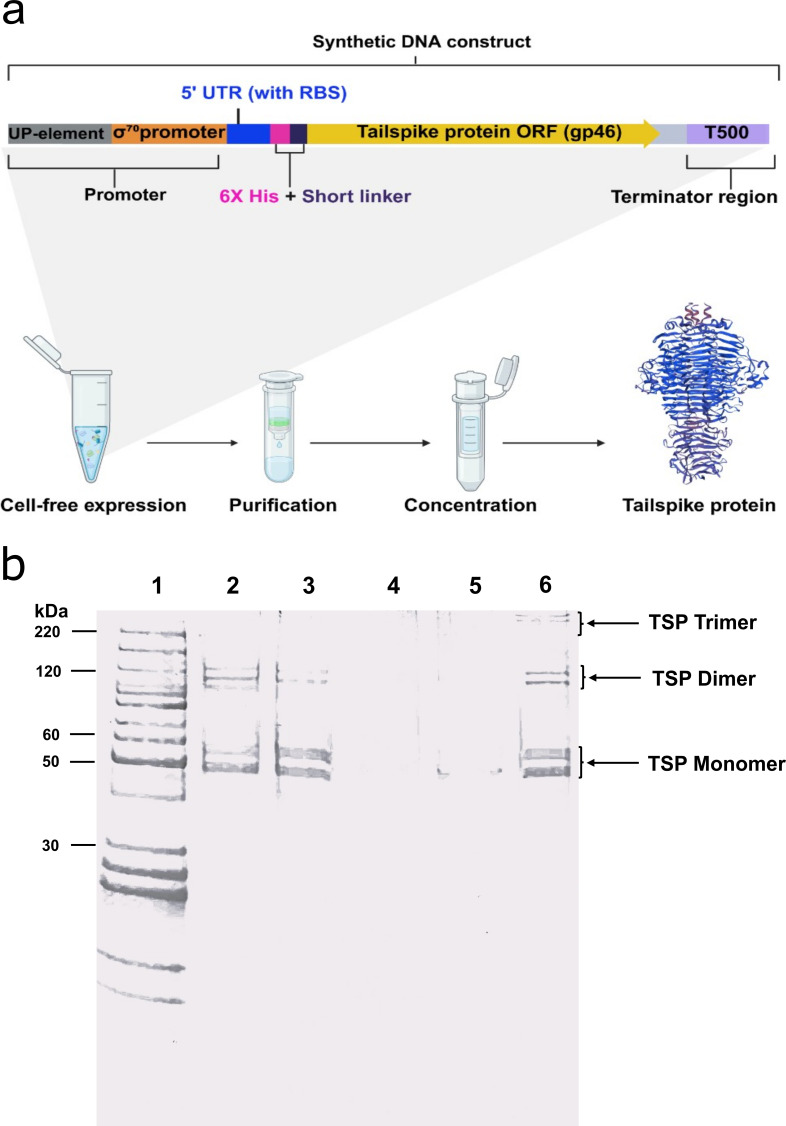
Cell-free expression and purification of SP6 TSP. (**a**) Graphical representation of the process for obtaining cell-free expressed SP6 TSP. The synthetic DNA construct used for TSP expression contains a sigma70 promoter region with UP element, 5’ UTR with ribosomal binding site (RBS), N-terminal 6x His-tag, followed by SP6 tailspike gene (*gp46*), and T500 terminator. The synthetic gene construct was expressed in an *E. coli-*based cell-free gene expression system. (**b**) SDS-PAGE analysis of purified TSP under various denaturing conditions. Lane 1: benchmark protein ladder; Lane 2: incubation at RT; Lane 3: boiled for 5 min; Lane 4: 6 M guanidine hydrochloride (gdmCl); Lane 5: GdmCl and sample boiling for 5 min; Lane 6: sample heat treatment at 72°C for 5 min. TSP monomers, dimers, and trimers are indicated.

We investigated whether the TSP from *Salmonella* phage SP6, a putative endo-rhamnosidase, could be used to enhance the infectivity of FO1. The SP6 TSP, encoded by gene *gp46* of *Salmonella* phage SP6, was produced using an *E. coli* cell-free transcription-translation (TXTL) expression system (Fig. 1a) (36, 37). SDS-PAGE analysis of purified TSP under various denaturing conditions revealed three distinct sets of bands (Fig. 1b). The lowest molecular weight bands were found to be around 50–60 kDa molecular weight and appeared to display the highest intensity compared to the other band pairs. This was consistent with the expected 60 kDa monomer size of SP6 TSP. The band pair around 120 kDa and higher molecular weight bands (>220 kDa) likely represent dimers and multimers, respectively. TSP is well known for its robust characteristics, such as trimeric structure and its strong internal hydrophobic interactions. These features are known to confer resistance to SDS denaturation, leading to altered electrophoretic mobility due to reduced SDS binding (38–40). Bioinformatically through sequence alignment, we observed that SP6 TSP has a conserved endo-rhamnosidase domain as previously reported for P22 TSP (Fig. S1a). Prior studies demonstrated that D393N and D396N mutations in the P22 TSP reduce catalytic turnover significantly (41, 42). Corresponding residues in SP6 (D280N and D283N) were mutated using site-directed mutagenesis two-step overlap extension PCR (43). A predicted 3D model of a SP6 monomer with highlighted catalytic domain and mutation sites was generated using Alphafold (Fig. S1b) (44). Functional assays were performed by supplementing purified SP6 TSP to tenfold serial dilutions of FO1 phage until a final concentration of 10 ng/µL, which were then spotted onto the bacterial host. It was observed that wild-type TSP significantly enhanced FO1 plaquing on *S*. Typhimurium 4/74, whereas the catalytic mutant (mTSP) exhibited reduced activity, confirming that the conserved domain is essential for enzymatic function and phage infectivity enhancement on this host (Fig. S1c).

### FO1 supplemented with SP6 TSP enhances infectivity on *Salmonella*

**Fig 2:**
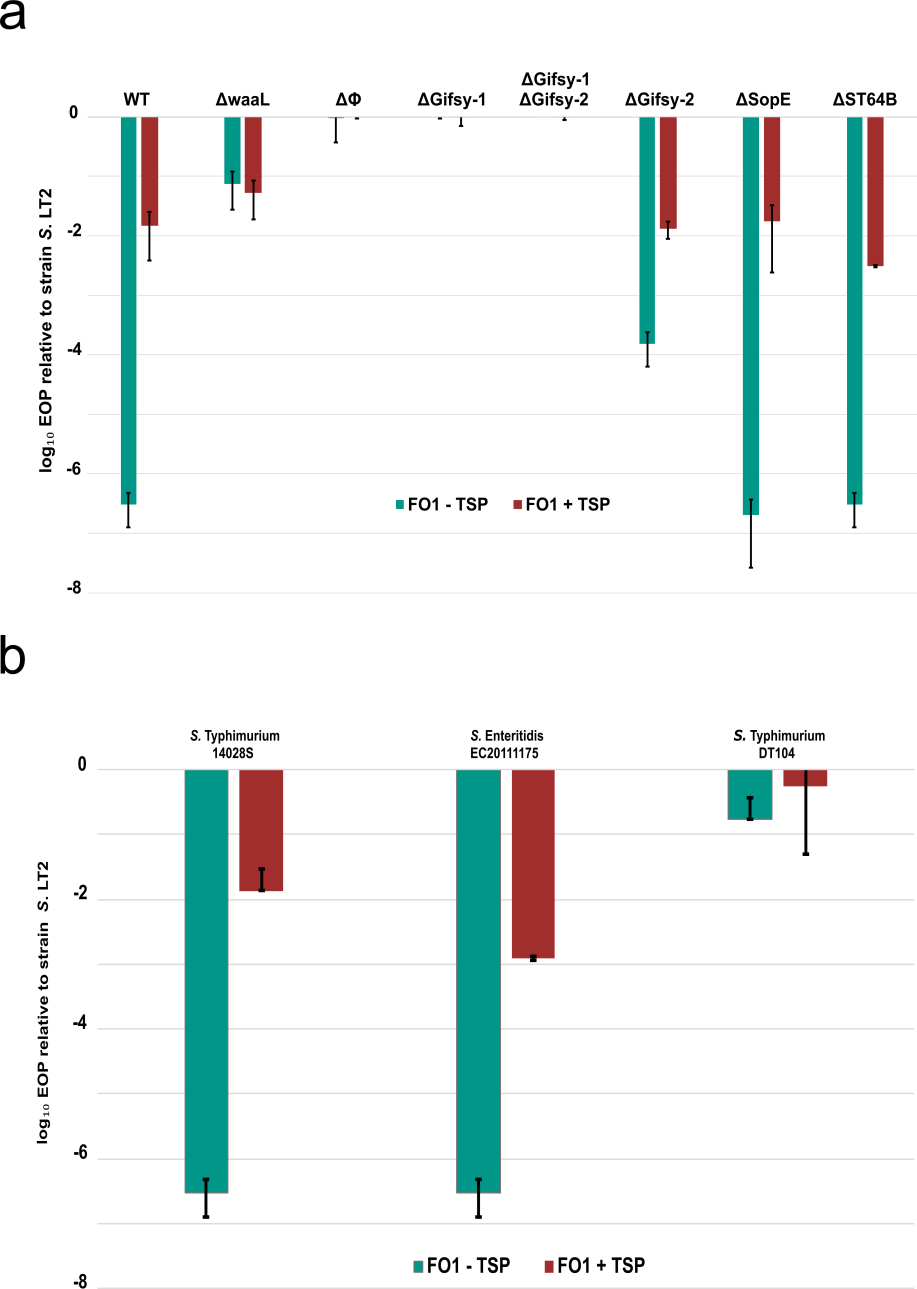
Supplementation of SP6 TSP enhances FO1 infectability across various *Salmonella* strains. (**a**) Log_10_ efficiency of plating (EOP) values of FO1 with TSP (green bar) and without supplemented TSP (red), infecting *S*. Typhimurium 4/74 wild type and various deletion strains. (**b**) Log_10_ EOP of FO1 ± supplemented TSP on *S*. Typhimurium ATCC 14028s, *S*. Enteritidis EC20111175, and *S*. Typhimurium DT104 hosts. Reference values were calculated for FO1 plaquing on *S*. Typhimurium LT2. Bars represent EOP values obtained from technical and biological triplicates, with error bars indicating standard deviations.

Purified SP6 TSP was supplemented to tenfold serial dilutions of FO1 phage to a final concentration of 10 ng/µL and spotted onto *S*. 4/74. It was observed that SP6 Gp46 TSP supplementation to FO1 increased EOP by approximately four logs on host *S*. 4/74 (Fig. 2a). To confirm whether the *S*. 4/74 O-antigen posed as a physical barrier for FO1 infection, a *S*. 4/74 Δ*waaL* deletion strain was constructed. This deletion strain is lacking the O-antigen ligase, which catalyzes the attachment of the O-antigen to the lipid A-core oligosaccharide (45). We observed that the EOP on *S*. 4/74 Δ*waaL* increased by five logs, confirming that *S*. 4/74 O-antigen serves as a physical barrier for FO1. Importantly, supplementation with SP6 TSP did not further enhance the EOP on the Δ*waaL* deletion strain, demonstrating that it functions to remove the O-antigen barrier to FO1 infection.

While the Δ*waaL* mutation increased FO1 infection on *S*. 4/74, it was observed that the EOP was still reduced compared to the permissive host *S. enterica* serovar Typhimurium LT2 (*S*. LT2). We considered the possibility that differences in the prophage repertoire between these strains may contribute to FO1 infection on *S. enterica*. Spot testing of FO1 on the prophage-cured strain, *S*. 4/74 ΔΦ, demonstrated EOP values close to 1, representing similar plaquing compared to *S*. LT2 (Fig. 2a). This suggested that one or more prophages in *S*. 4/74 confer resistance to FO1. To identify the responsible element(s), we tested a panel of *S*. 4/74 strains with individual prophages deleted. It was observed that the deletion of prophage Gifsy-1 restored EOP values of FO1 on test strain *S*. 4/74 compared to reference strain *S*. LT2 (Fig. 2a). The deletion of prophage Gifsy-2 increased the EOP of FO1 by approximately two logs. Meanwhile, the removal of prophages SopE and ST64B had no discernible effect. Interestingly, the FO1 plaque sizes were also increased on *Salmonella* strains where Gifsy-1 had been removed, compared to *S*. 4/74 Δ*waaL or S*. 4/74 supplemented with TSP (Fig. S2).

To test this phenotype in other genetic backgrounds, *S. enterica* serovar Typhimurium ST313 strain D23580 (*S*. D23580) and *S. enterica* serovar Typhimurium strain ATCC 14028s (*S*. 14028s) were tested (46, 47). Despite sharing similar prophage regions with *S*. 4/74 including Gifsy-1, FO1 did not show reduced EOP on *S*. D23580 (Fig. S3 and S4). Prophage-cured strains of *S*. D23580 that were subsequently lysogenized with either P22 (*S*. D23580 ΔΦ(P22)) or BTP1 (*S*. D23580 ΔΦ(BTP1)), both of which contain known *gtrABC* O-antigen modifying gene clusters, did not affect FO1 infectability (48, 49). However, similar restrictive phenotypes to that of *S*. 4/74 were observed for *S*. 14028s and *S. enterica* serovar Enteritidis strain EC20111175. For the wild-type strain *S*. DT104, FO1 infectivity showed an EOP reduction of one log, while supplementation of SP6 TSP increased the EOP to approximately one (Fig. 2b).

### Gifsy-1 prophages display large diversity in their non-structural encoding regions

**Fig 3:**
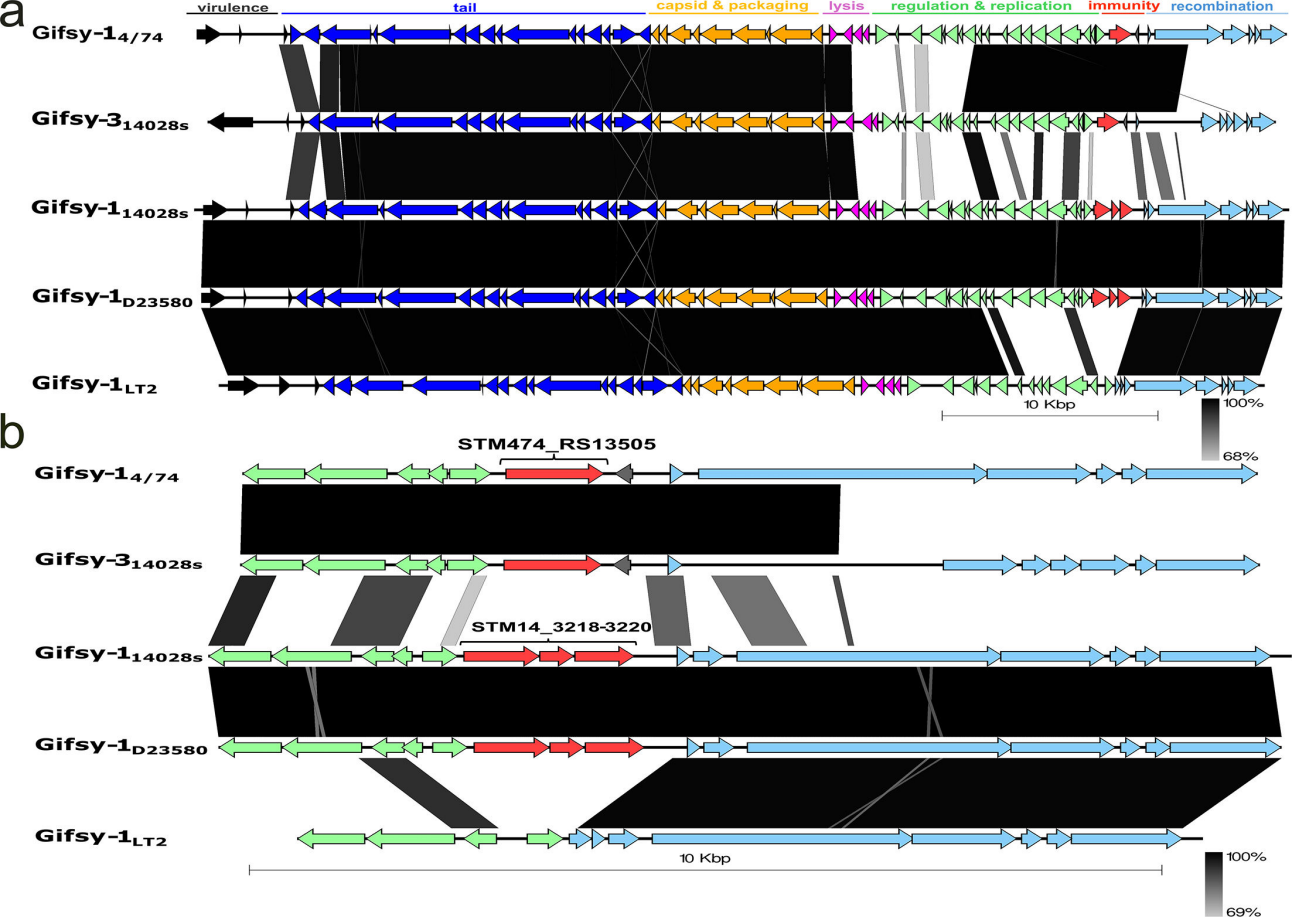
Comparative genomic analysis reveals high variability in the Gifsy prophage regions of different *Salmonella* Typhimurium strains. (**a**) Linear genome alignment of Gifsy prophage regions found in *S*. Typhimurium 4/74, *S*. Typhimurium 14028s, *S*. Typhimurium D23580, and *S*. Typhimurium LT2. Prophage subregions are marked according to their predicted functional categories: Virulence gene cluster (gray), phage tail associated genes (dark blue), capsid and packaging gene cluster (orange), lysis cassette (purple), regulation and replication (green), immunity (red), recombination (light blue), and unknown (gray). (**b**) Zoom-in on highly variable prophage region including regulation, immunity, and recombination subregions. Pairwise homology between genes is indicated by black shading, ranging from 69% (light gray) to 100% (black). Scale bar indicates a nucleotide length of 10 kb pairs.

The different FO1 EOP values across different Gifsy-1 containing *Salmonella* strains prompted further exploration of the Gifsy-1 regions (Fig. 3a). Prophage immunity regions are known to frequently harbor antiviral defense systems (48). It was observed that *S*. 4/74 contains a Gifsy-1 immunity region that closely resembles one Gifsy-3 prophage region in *S*. 14028s (Genbank accession: NC_016856.1, coordinates 1285764–1336058) (Fig. 3a). This observation of Gifsy-1 and Gifsy-3 sharing specific sequences has been reported previously (24).

In addition to Gifsy-3, *S*. 14028s also contains a Gifsy-1 prophage region located between coordinates 2780105 and 2830876 (24). Gifsy-1_14028s_ contains a gene cluster (locust tag: STM14-3218_3220) in its immunity region that has recently been revealed to encode for a novel anti-phage defense mechanism (Fig. 3b). This system, named remAIN, has been shown to block lytic cycles of co-residing prophages as well as incoming foreign phages (49). We observed that there was no homology between the *remAIN* gene cluster and the immunity region found in Gifsy-1_4/74_. In contrast, it was shown that Gifsy-1_D23580_ and Gifsy-1_14028s_ are identical in this region. Thus, *S*. D23580 also contains a *remAIN* gene cluster. Instead of the *remAIN* three-component gene cluster, both Gifsy-1_4/74_ and Gifsy-3_14028s_ contain a single gene in their immunity region (locus tag: STM474_RS13505; GenBank accession: CP000603.1, coordinates: 2,768,985–2,770,070).

### Gifsy-1_4/74_-encoded immunity gene confers resistance against FO1

**Fig 4:**
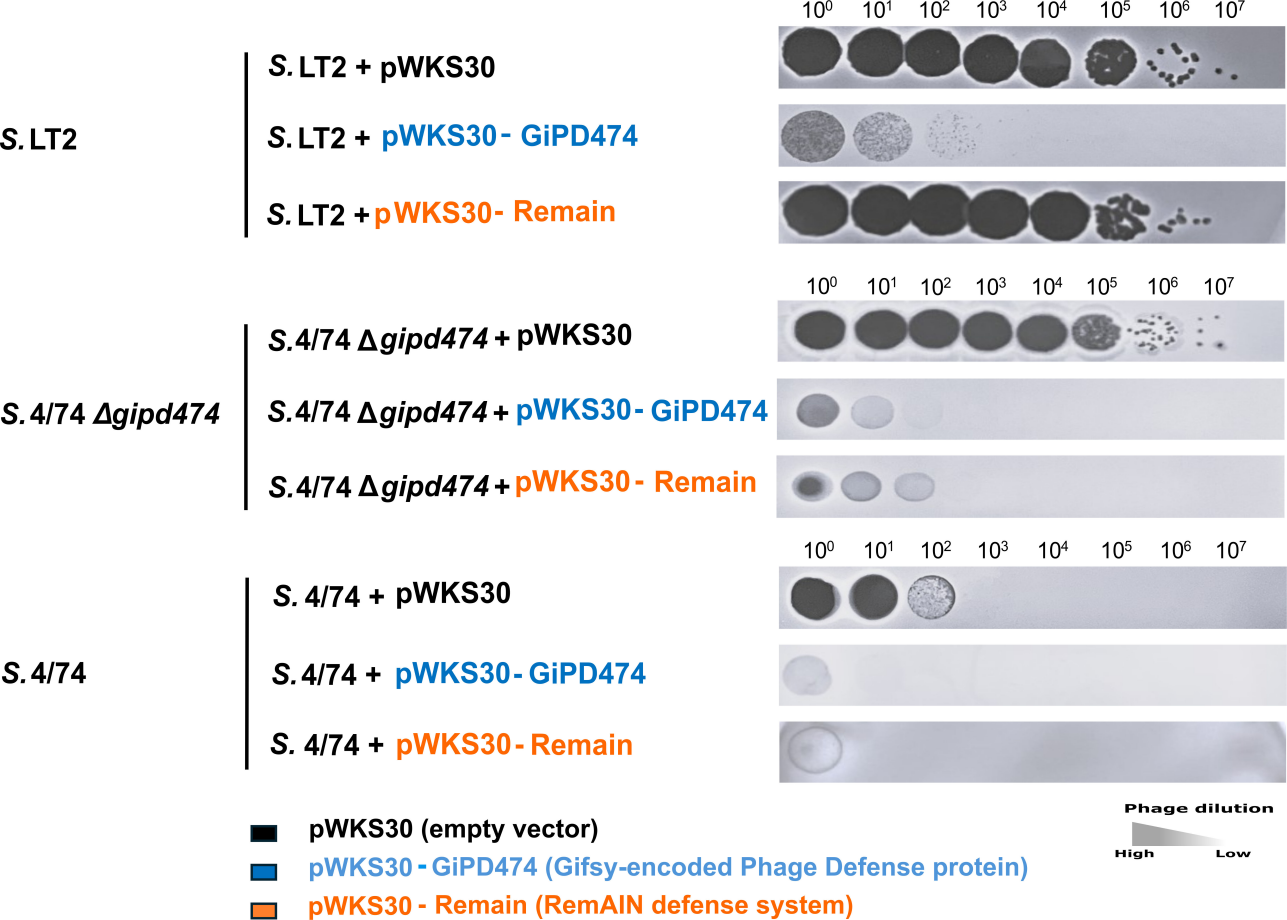
Genetic deletion and complementation confirm the Gifsy-encoded phage defense protein (*GiPD474*) confers resistance to phage FO1 infection. Three different *S. enterica* genetic backgrounds were complemented with two different plasmids containing two different Gifsy-1 encoded phage immunity proteins. Reference strain *S*. Typhimurium LT2 containing low copy cloning vector pWKS30 was used as a negative control. The recently discovered remAIN system was used as a positive control for resistance against FO1. Tenfold dilutions of FO1 were spotted onto a bacterial lawn to examine its phage sensitivity.

Further investigation was focused on the involvement of gene STM474_RS13505 during FO1 infection in *Salmonella*. Two expression plasmids were constructed using a low-copy-number expression vector pWKS30 with a pLac promoter driving gene expression: one containing gene STM474_RS13505 from *S*. 4/74, hereafter named *gipd474* (Gifsy-encoded phage defense protein from strain 474), and another containing the *remAIN* gene cluster (pWKS30-Remain) from *S*. 14028s. Subsequently, the two expression plasmids and one empty vector were transformed into strains *S*. LT2, *S*. 4/74, and *S*. 4/74 Δ*gipd474*. On *S*. LT2, it was observed that pWKS30-GiPD474 caused a significant reduction in FO1 plaquing compared to the empty vector, whereas remAIN did not have an effect in this genetic background (Fig. 4). For the deletion strain *S*. 4/74 Δ*gipd474*, we observed that FO1 EOP values were restored compared to that on the permissive host *S*. LT2. After genetic complementation of *S*. 4/74 Δ*gipd474* with pWKS30-GiPD474, it was shown that FO1 resistance was reestablished, as was similarly exhibited for pWKS30-Remain. Transformed *S*. 4/74 strains with pWKS30-GiPD474 and pWKS30-Remain showed a further reduction in FO1 plaque-forming units compared to the complemented strains *S*. 4/74 Δ*gipd474*, confirming that Gifsy-1–encoded gene *gipd474* can play a major role in FO1 resistance in *Salmonella*.

### GiPD474 strongly reduces the infectivity of contractile tailed phages

**Fig 5:**
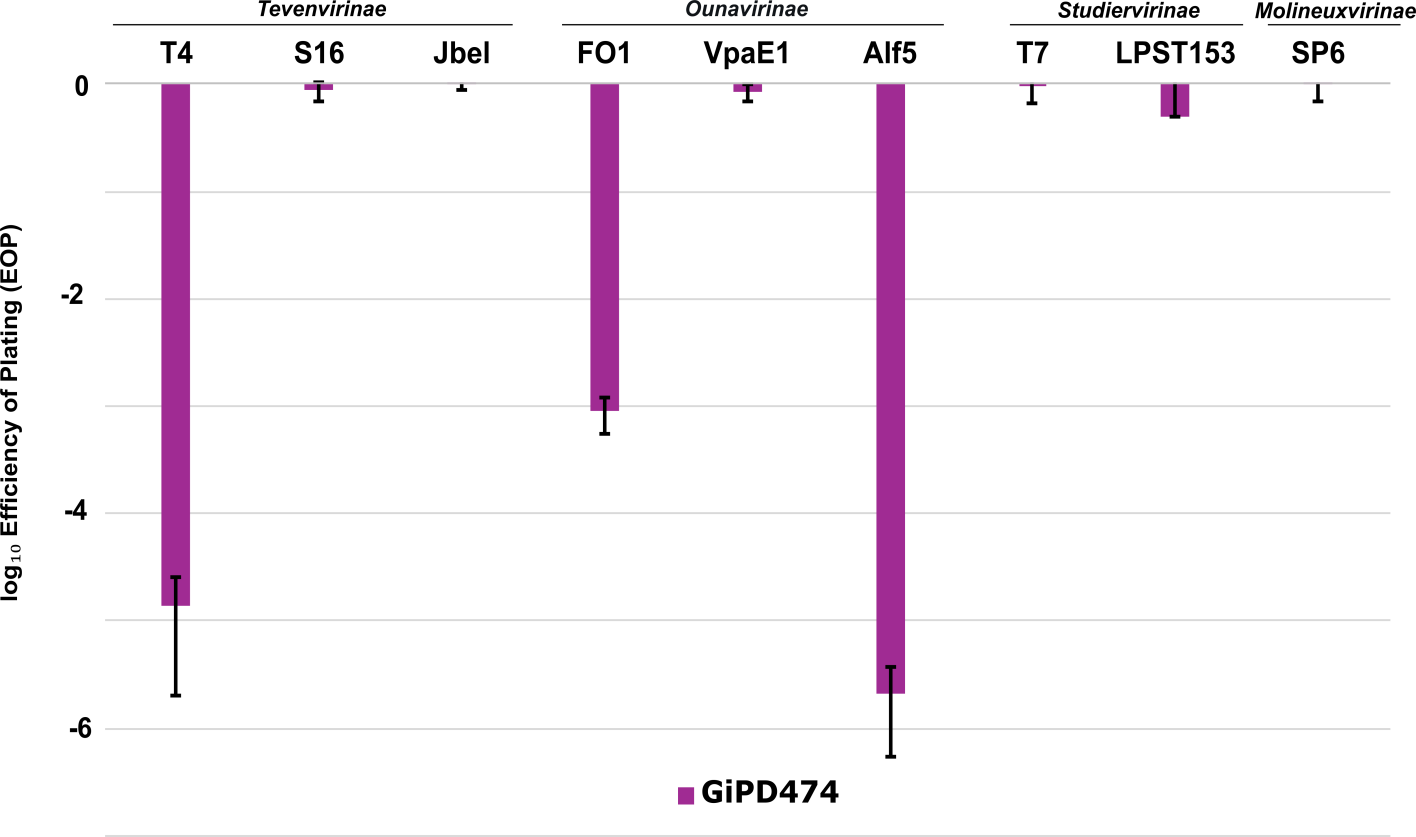
GiPD474 protein provides protection against various tailed phages. Log_10_ efficiency of plating values was calculated and plotted for various *Salmonella* and coliphages. Reference strains *S*. Typhimurium LT2 and *E. coli* K-12 contained the pWKS30 cloning vector. Test strains *S*. Typhimurium LT2 and *E. coli* K-12 contained plasmid pWKS30-GiPD474. Bars represent EOP values obtained from technical and biological triplicates, with error bars indicating standard deviation. Phages are grouped according to their respective virus subfamily.

The target range of the novel antiviral defense protein was assessed by challenging it with phages from diverse phage families. Some selected phages include T4, T7, and SP6, which are some of the most well-studied phages (50–52). Phages S16 and FO1 are both currently being used in a commercial phage cocktail to reduce *Salmonella* in food production processes (53). Both Alf5 and VpaE1 are more recently discovered coliphages that are highly related to FO1 and belong to the same phage subfamily of the *Ounavirinae* (54, 55). Additionally, two more recently discovered phages were included: Jbel and LPST153. Polyvalent phage Jbel infects *Salmonella*, *E. coli*, and *Shigella* and belongs to the sub-family of *Tevenvirinae* (56). Lastly, *Salmonella* phage LPST153, which is closely related to T7 phage, belongs to the *Studiervirinae* (57). It was observed that, besides FO1, both Alf5 and T4 phages showed a strong reduction in their EOP when exposed to GiPD474. Phage T4 showed a 5-log reduction, whereas Alf5 exhibited an EOP reduction of approximately 5.5 logs (Fig. 5). The other tested phages (T7, SP6, LPST153, Jbel, VpaE1, and S16) were unaffected, demonstrating that GiPD474 is only functional against certain phages (Fig. 5; Fig. S5).

### GiPD474 contains an ATPase domain, and prophage-associated homologs are widespread across *Enterobacteriaceae*

**Fig 6:**
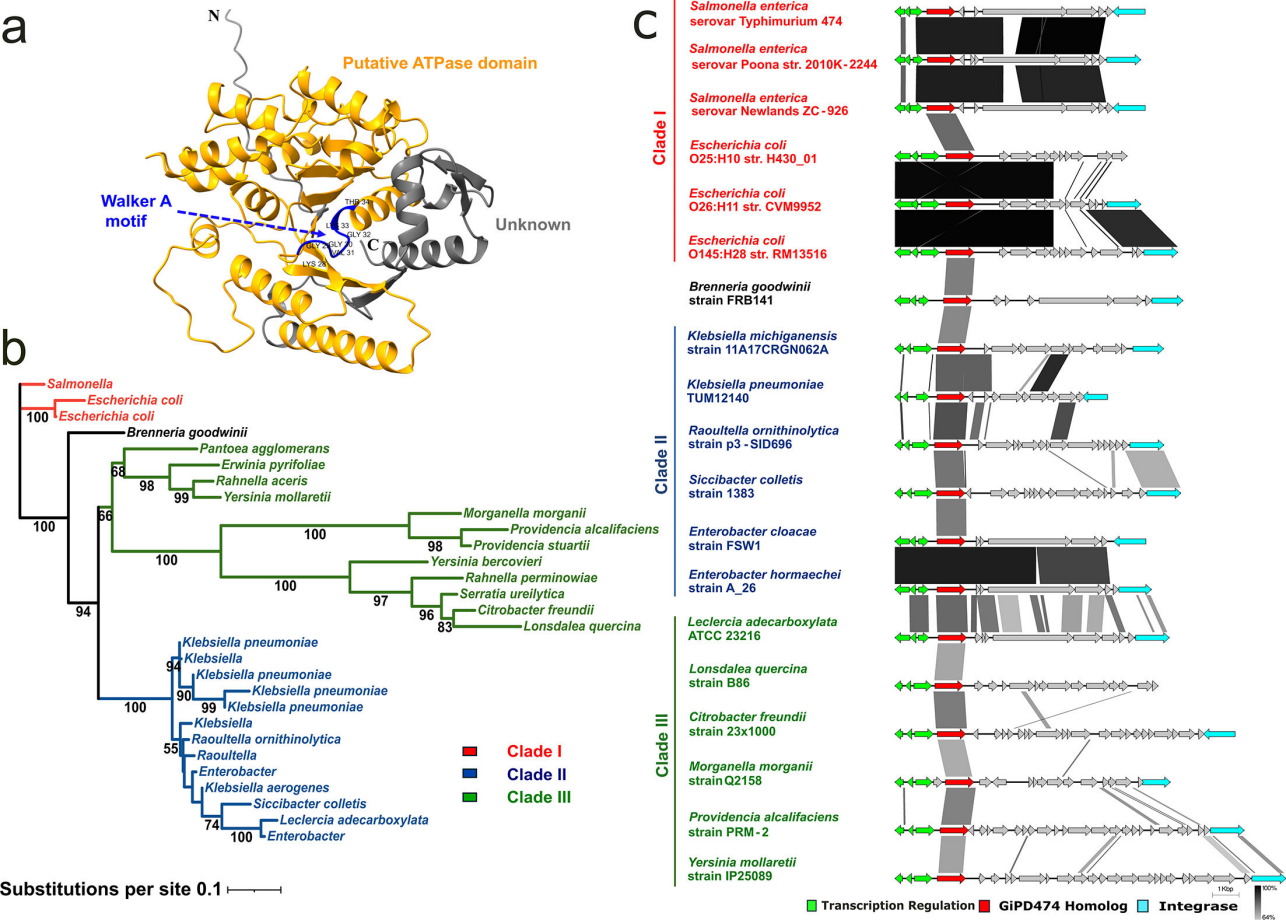
Phylogenetic analysis and gene neighborhood comparison of GiPD474 reveal its distribution across diverse members of the *Enterobacteriaceae* family. (**a**) Predicted 3D protein structure of GiPD474 was generated displaying AAA domain (orange), Walker A motif (blue), and unknown domain (gray). (**b**) Maximum likelihood phylogenetic tree of GiPD474 across members of the *Enterobacteriaceae* bacterial family. Three major clades were defined: Clade I (red), predominantly *Salmonella* and *Escherichia;* Clade II (blue), composed largely of clinically relevant *Klebsiella* and *Enterobacter* species; and Clade III (green), plant and environment-associated pathogens. (**c**) Gene neighborhood comparison of GiPD474 homologs displayed in different members of the *Enterobacteriaceae* family. GiPD474 homologs (red) are flanked by transcription regulation modules (green) and downstream integrase (light blue). Pairwise homology between genes is indicated by black shading ranging from 69% (light gray) to 100% (black).

A predicted 3D model was generated from the GiPD474 protein sequence using Alphafold (44). The 3D-generated model was then used to make structural predictions, and the DALI server was used to search for structural homologs (58). Strong matches were defined as those with >20% sequence identity. The top 150 structural hits all showed Z-scores above 20, indicating substantial structural similarity (Table S3). These matches primarily aligned with residues 29–275 of GiPD474, encompassing a region that includes a glycine-rich motif GGVGKT between residues 29 and 34, a hallmark of the Walker A motif (GXXXXGKT/S) (59). This motif is characteristic of the phosphate-binding loop (P-loop) found in many NTPases. Additionally, most matches are proteins with known ATPase activity, such as ParA, MinD, and Soj (60, 61). Finally, to complement the structural analysis, InterProScan identified GiPD474 as a P-loop-containing nucleoside triphosphate hydrolase and annotated both an AAA_31 domain (IPR000924) and a ParAB family region (IPR027417) spanning residues 23–288 (60). Together, these structural and domain-based bioinformatic predictions strongly support that GiPD474 encodes a putative AAA + ATPase (Fig. 6a).

Phylogenetic analysis of GiPD474 homologs revealed three major clades: Clade I (e.g., *Salmonella* and *E. coli*), Clade II (e.g., *Klebsiella*, *Enterobacter*), and Clade III (e.g., *Pantoea*, *Yersinia*, *Providencia*) (Fig. 6b). Strong bootstrap values support divergence among the different clades.

Gene neighborhood comparisons showed that GiPD474 homologs are consistently located near putative transcription regulator modules, which include a putative CII family transcription regulator, a putative Cro-type transcription regulator, and a putative phage repressor (Fig. 6c) (62). Further downstream of the GiPD474 homologs, integrase-encoding genes were found across almost all clades.

GiPD474 homologs, integrase-encoding genes, were found across almost all clades. The widespread distribution, together with its prophage-encoded region, strongly supports horizontal gene transfer as a mechanism for its divergence across different members of the *Enterobacteriaceae* family.

### High transcription levels of *gipd474* were found during the exponential growth phase and multiple stress conditions of *S*. Typhimurium 4/74

Using the SalComD23580 gene expression compendium, we analyzed the expression profile of gene *STM474_2742* (old locust tag of *gipd474*) in *S*. 4/74 (Fig. S6) (62). The gene exhibited strong expression (>500 transcripts per million) during the early exponential phase (EEP), mid-exponential phase (MEP), and late exponential phase (LEP) growth stages. Gene expression was also strongly upregulated during stress-induced conditions, including anaerobic shock, NaCl, bile, and iron depletion as well as under certain SPI2 (*Salmonella* Pathogenicity Island 2) inducing conditions, such as nitric oxide shock. The gene expression was observed to be moderate during SPI2-inducing conditions, such as peroxide shock and in macrophages, and showed low expression after entering the late stationary phase (LSP) (49 TPM).

## DISCUSSION

In this study, we identified a novel prophage-encoded gene in *S*. 4/74 that restricts plaque formation by multiple tailed phages. Located in the immunity region of the Gifsy-1 prophage, this single gene *gipd474* represents a previously uncharacterized anti-phage defense mechanism. Unlike known multi-component systems such as *remAIN*, this system consists of only one gene that confers robust phage resistance. Our findings expand the catalog of prophage-encoded antiviral systems and highlight the functional diversity within the immunity regions of temperate phages.

We further demonstrated that the SP6 TSP can be expressed in an *E. coli*-based cell-free system and retains its catalytic function. SP6 TSP shares its catalytic domain with the well-characterized P22 TSP, which is essential for its enzymatic activity. On SDS-PAGE gel, the purified TSP appeared as multiple bands, consistent with its trimeric structure and strong hydrophobic interactions. The high structural stability of TSP makes it a promising candidate for incorporation into FO1 formulations to enhance adsorption efficiency and broaden the host range in therapeutic and food safety applications.

We observed that when supplemented with 10 ng/µL SP6 Gp46 TSP, phage FO1 plaquing was enhanced on *S*. 4/74. After deletion of the *waaL* gene, which encodes the O-antigen ligase, an increased EOP was observed, suggesting a possible steric hindrance of phage FO1 by the O-antigen of *S. 4/74*. However, the EOP remained reduced compared to the *S*. LT2 host. This suggests that GiPD474 possibly acts at a post-adsorption stage of the phage infection cycle. However, further experimental work is needed to confirm this mode of action.

Comparative analysis of Gifsy-1-encoded defense systems reveals interesting distinctions between remAIN and GiPD474. The remAIN system did not restrict FO1 in the *S*. LT2 background, suggesting that remAIN requires host-specific cofactors for functionality. Interestingly, FO1 did not show a significantly reduced EOP on *S*. D23580, even though a copy of the remAIN gene cluster is harbored within its Gifsy-1 prophage. Previous research has shown that a specific mutation in the promoter region of Gifsy-1 in *S*. D23580 renders the prophage defective (47). It is plausible that this mutation also affects the expression of the remAIN system, which would explain the lack of FO1 resistance observed in *S*. D23580. This is consistent with our findings on *S*. DT104, which was also found to be a permissive host for FO1, as it has been previously reported that certain US lineages of *S*. DT104 lack the Gifsy-1 prophage altogether (63).

Conversely, GiPD474 maintained its antiviral effect in *S*. LT2 and even in heterologous hosts such as *Escherichia coli*. This suggests that GiPD474 may function independently of host-specific factors, which could explain its widespread conservation across *Enterobacteriaceae*. Consistently, we identified GiPD474 homologs within both Gifsy-1 and Gifsy-3 prophages, consistent with extensive gene exchange between these elements. Prior studies have proposed that such recombination events, likely mediated by intrachromosomal conversion, facilitate the diversification of *Salmonella* prophages (22, 63, 64).

Phylogenetic analysis further demonstrated that GiPD474 is broadly distributed among distantly related bacterial species and consistently localized within prophage regions. This strongly supports its dissemination via horizontal gene transfer (HGT) (65). Kieffer et al. (66) further showed that, in addition to prophages, mobile integrons in *E. coli* also encode a diverse array of phage defense systems, reinforcing the idea of highly mobile, cost-efficient defense islands (66).

We found that the GiPD474 protein contains a characteristic Walker A motif and an AAA domain and shares homology with other ATPase-containing proteins. Recent studies have shown that certain single-module ATPase-containing proteins can mediate phage resistance in *E. coli* and *Salmonella*. One such example is the AVAST (antiviral ATPases/NTPases of the STAND superfamily) type II (Avs2), a member of the STAND NTPase superfamily (14). In a study by Gao et al. (67), an *E. coli*-derived Avs2 protein was shown to recognize its viral target via a C-terminal sensor domain. Upon recognition, ATP-dependent trimerization of the ATPase domain activated a toxic N-terminal effector, thereby halting phage replication. Interestingly, Avs families 1–3 were found to target the phage large terminase subunit, whereas Avs4 instead recognized the phage portal (67).

A similar Avs2-family protein was identified in *K. pneumoniae*, where it protected bacterial populations via abortive infection against phage by binding the large terminase subunit. Additionally, it also appeared to bind a phage protein Ksap1 with unknown function (68). More broadly, other STAND family NTPase proteins were also found to mediate antiviral activity in different defense systems (69). These systems appear to operate as modular switches: upon phage infection, ATP binding induces conformational changes that drive oligomerization and activate toxic effectors such as nucleases, hydrolases, proteases, nucleosidases, or other unknown protein functions (70). The precise mode of action of this novel anti-phage defense system is still unknown, and further investigation needs to be done to elucidate its exact function.

The high expression levels of GiPD474 during growth and under stress conditions are characteristics of several defense systems. While constitutive expression enables rapid phage response, it may also impose a fitness cost, reflecting a trade-off between broad protection and the risk of autoimmunity (71). Different defense systems, such as cyclic oligonucleotide-based bacterial anti-phage signaling systems (CBASS), restriction–modification (R–M) systems, clustered regularly interspaced short palindromic repeat-Cas (CRISPR-Cas) system, and bacteriophage control infection (BCI), were found to be regulated by quorum sensing (QS) (72). In contrast, other defense systems, such as Tail assembly blocker (Tab) and NLR-related protein bNACHT01, are constitutively expressed (69, 73). However, a direct experiment measuring the expression of GiPD474 in response to phage infection would be crucial to confirm its anti-phage role and to determine if there is an inducible component to its regulation. This remains a key area for future investigation.

In conclusion, we demonstrate that FO1 can overcome O-antigen–mediated barriers in *S*. Typhimurium 4/74 through SP6 TSP supplementation, thereby uncovering a Gifsy-encoded novel antiviral defense gene. This previously uncharacterized protein provides broad protection against diverse tailed phages and is widely conserved across *Enterobacteriaceae*. Its prophage association and broad conservation suggest a major role in bacterial immunity. Understanding such systems is essential for predicting phage resistance and developing effective phage-based strategies for clinical therapeutics, food safety, agriculture, and animal health.

## MATERIALS AND METHODS

### Bacterial strains, plasmids, and culture conditions

All bacterial strains, phages, and plasmids used in this study are presented in Table S1 (74–82). All strains were grown in Lennox broth (LB: 10 g/L tryptone, 5 g/L yeast extract, 5 g/L NaCl) and incubated at 37°C with shaking (200 rpm) under aerobic conditions. Kanamycin (50 µg/mL), ampicillin (100 µg/mL), and IPTG (1 mM) were added as required.

### Bacteriophage propagation and spot assay

All phages were stored in phage buffer (10 mM Tris-HCl pH 7.5, 10 mM MgCl_2_, 68 mM NaCl, and 1 mM CaCl_2_) and 25% glycerol stocks were stored at −80°C. *Salmonella* phage FO1 was propagated using *S*. Typhimurium LT2 as its host and purified using a cesium chloride gradient (39). *Salmonella* phages S16, SP6, and LPST153 were propagated using bacterial host *S*. Typhimurium LT2. Coliphages T4, T7, Jbel, and Alf5 were propagated using bacterial host *Escherichia coli* (*E. coli*) K-12 as their host and phage VpaE1 on host *E. coli* B^E^. Phage lysates were prepared using a plate lysis technique as described (83). Spot assays were carried out by preparing 1.5% LB agar plates and 0.5% soft LB agar layer containing an overnight bacterial culture. Both LB agar layers contained 5% glycerol and 1% Coomassie Brilliant Blue G-250 for better visualization of plaque-forming units (PFU). Phage solutions were serially diluted tenfold in phage buffer and spotted onto their respective bacterial hosts. Plates were incubated overnight under optimal culture conditions. The infectivity of a bacteriophage on a given host was quantified by determining efficiency of plating (EOP). This was determined by calculating the ratio of plaque formation obtained on certain test strains over plaque formation on the reference strains *S*. Typhimurium LT2, *E. coli* K-12, and *E. coli* B^E^. All EOP values in this study were calculated from technical and biological triplicates.

### Genetic manipulations

Synthetic DNA constructs were generated by PCR amplification of the SP6 tailspike gene (*gp46*) using KOD One PCR Master Mix (Millipore Sigma, USA) and gene-specific primers (primer sequences listed in Table S2). The product was re-amplified with primers encoding a 5′ His-tag (round 2), followed by primers adding a 5′ UTR and P70a promoter (round 3), and finally with primers introducing a 5′ UP-element and 3′ T500 terminator (round 4). Site-directed mutagenesis of the SP6 TSP gene was performed using two-step overlap extension PCR (OE-PCR) to introduce point mutations into *gp46*, generating mTSP DNA constructs (43). Genetic deletions in *Salmonella* were made using λ Red recombination using the insertion of a kanamycin resistance marker from pKD4 (84). PCR fragments were cleaned up and concentrated using DNA Clean & Concentrator-5 (Zymo Research, USA). For genetic complementation, genes of interest were PCR amplified using KOD One PCR Master Mix (Millipore Sigma, USA) and inserted into the low-copy-number plasmid pWKS30 with an ampicillin resistance marker (85). Cloning was performed using the NEBridge Golden Gate Assembly Kit with BsmBI-v2 restriction enzyme (New England Biolabs, USA). Circularized plasmids were transformed into competent *E. coli* DH5α via heat shock.

Transformants were grown on selective LB plates containing 100 µg/mL ampicillin. Individual colonies were picked and grown overnight in LB liquid culture supplemented with 100 µg/mL ampicillin. Plasmid DNA was extracted using E-Z 96 FastFilter Plasmid Kit (Omega Bio-tek, USA). Successful insertion of genes of interest into cloning vector pWKS30 was confirmed by whole plasmid sequencing. Electrocompetent *S*. LT2 and *E. coli* K-12 strains were transformed with empty vector pWKS30, pWKS30-GiPD474, and pWKS30-Remain using MicroPulser electroporation apparatus (Bio-Rad, USA) and selectively grown on LB plates containing 100 µg/mL ampicillin. Expression of pWKS30 plasmids in *E. coli* K-12 was induced by supplementation with 1 mM IPTG in a soft 0.5% LB agar layer.

### Tailspike protein expression and purification

The wild-type and mutant SP6 TSPs were expressed using an *E. coli*-based TXTL cell-free system (36, 37). Cell-free extract reactions were supplemented with 10 nM of the synthetic DNA construct encoding the His-tagged TSP and incubated at 29°C overnight in 1.7 mL microcentrifuge tubes. Reactions were diluted with 90 µL phage buffer and purified using 0.2 mL HisPur Cobalt Spin Columns (Thermo Scientific, USA). Eluted proteins were dialyzed with phage buffer using Amicon Ultra centrifugal filter units with a 30 kDa molecular weight cutoff (Merck Millipore, USA). Protein concentration was measured using the standard Bradford assay (Thermo Scientific, USA). Purified protein samples (TSP and mTSP) were analyzed using SDS-PAGE. Samples were mixed with 4× Laemmli sample buffer (Bio-Rad, USA) containing 100 mM DTT and denatured under varying conditions: either by heating at 72°C or 95°C for 5 min or by adding 6 M guanidine hydrochloride (GdmHCl). Approximately 1 µg of each protein sample was loaded onto a precast Tris-Glycine 4–20% gradient gel (MP Biomedicals, USA). Electrophoresis was performed in 1× Tris-Glycine SDS running buffer at 150 V for 60 minutes. Gels were stained with QC Colloidal Coomassie Stain (Bio-Rad, USA) according to the manufacturer’s protocol and destained in ultrapure water. Protein molecular weights were estimated using the BenchMark Protein Ladder (Thermo Fisher Scientific, USA).

### Bioinformatics and phylogenetics

Multiple sequence alignments of Gifsy prophage regions and gene neighborhood comparison analysis of *gipd474* homologs were generated using Easyfig version 2.2.5 (86). Homologs of the GiPD474 protein were identified using BLASTP (NCBI) against the RefSeq Select protein database with parameters: e-value of 1e-5 and BLOSUM62 scoring matrix. Hits were filtered to retain sequences with ≥70% identity and ≥70% query coverage. Redundant sequences were clustered using CD-HIT at a 95% identity threshold (87). Multiple sequence alignments of GiPD474 homologs were performed using MAFFT v7.490 using the L-INS-i algorithm for high-accuracy local pairwise alignment (88). A maximum likelihood phylogenetic tree was built using IQ-TREE v2.4.0 with ModelFinder Plus to automatically select the best-fit substitution model (89). Branch support was assessed with 1,000 ultrafast bootstrap replicates. Tree visualization and annotation were carried out using iTOL v7 (90).

### Protein 3D structure prediction

Three-dimensional structure predictions of the TSP and GiPD474 were generated using AlphaFold2, through the ColabFold notebook (91), and structural homology was assessed using Dali server (58). The amino acid sequences were submitted via the web-based ColabFold v1.5.5 interface using default parameters, including MMseqs2 for multiple sequence alignment generation. Structural figures were visualized in UCSF ChimeraX version 1.1 (92).

### DNA sequencing

All constructed plasmids and synthetic DNA constructs have been sequenced to confirm the correct nucleotide sequences. Whole plasmid sequencing and linear PCR sequencing were performed by Plasmidsaurus using Oxford Nanopore Technology, followed by custom analysis and annotation.

## Data Availability

Genomes of all bacteriophages used in this study, including Felix O1 (NC_005282.1), S16 (NC_020416.1), SP6 (NC_004831.2), LPST153 (MK907285.1), Jbel (PP942088.1), Alf5 (NC_031082.1), VpaE1 (NC_027337.1), T4 (NC_000866.4) and T7 (NC_001604.1), are publicly available in the NCBI database. The STM474_RS13505 (GiPD474) amino acid sequence can be found in the NCBI Protein database using accession number WP_000368620.1. All other relevant data are available within the article and supplemental material or upon request.

## SUPPLEMENTAL MATERIAL

Figure S1 aem.01384-25-s0001.tif Catalytic domain mutations in SP6 TSP reduce FO1 infectivity.

Figure S2 aem.01384-25-s0002.tif Plaque assay SP6 TSP enhancing infectivity of phage FO1 across diverse *Salmonella* strains.

Figure S3 aem.01384-25-s0003.tif Supplementation of SP6 TSP does not enhance FO1 infectivity on *S*. Typhimurium D23580 or its prophage deletion mutants.

Figure S4 aem.01384-25-s0004.tif Plaque assay SP6 TSP enhancing infectivity of phage FO1 on *S*. Typhimurium D23580, its prophage deletion strains, and lysogenized strains.

Figure S5 aem.01384-25-s0005.tif Spot assay demonstrates that GiPD474 confers resistance to diverse lytic bacteriophages onto bacterial hosts S. Typhimurium LT2 and *E. coli* K-12.

Figure S6 aem.01384-25-s0006.tif Absolute and relative expression of STM474_2742 (*gipd474*) in *Salmonella* Typhimurium 4/74 under various growth and stress conditions obtained from the SalComD23580 transcriptome compendium.

Supplemental legends aem.01384-25-s0007.docx Legends for Fig. S1 to S6.

Table S1 aem.01384-25-s0008.docx Strains and plasmids used in this study.

Table S2 aem.01384-25-s0009.docx Primers used in this study.

Table S3 aem.01384-25-s0010.xlsx Top DALI server hits for GiPD474.

ASM does not own the copyrights to Supplemental Material that may be linked to, or accessed through, an article. The authors have granted ASM a non-exclusive, world-wide license to publish the Supplemental Material files. Please contact the corresponding author directly for reuse.
