# Supplementary figures and images for: A Gifsy prophage-encoded protein confers broad phage resistance in Salmonella enterica and is widely distributed across Enterobacteriaceae
Source: Appl Environ Microbiol. 2025 Nov 10;91(12):e01384-25. doi: 10.1128/aem.01384-25 (PMC12724215; doi:10.1128/aem.01384-25)

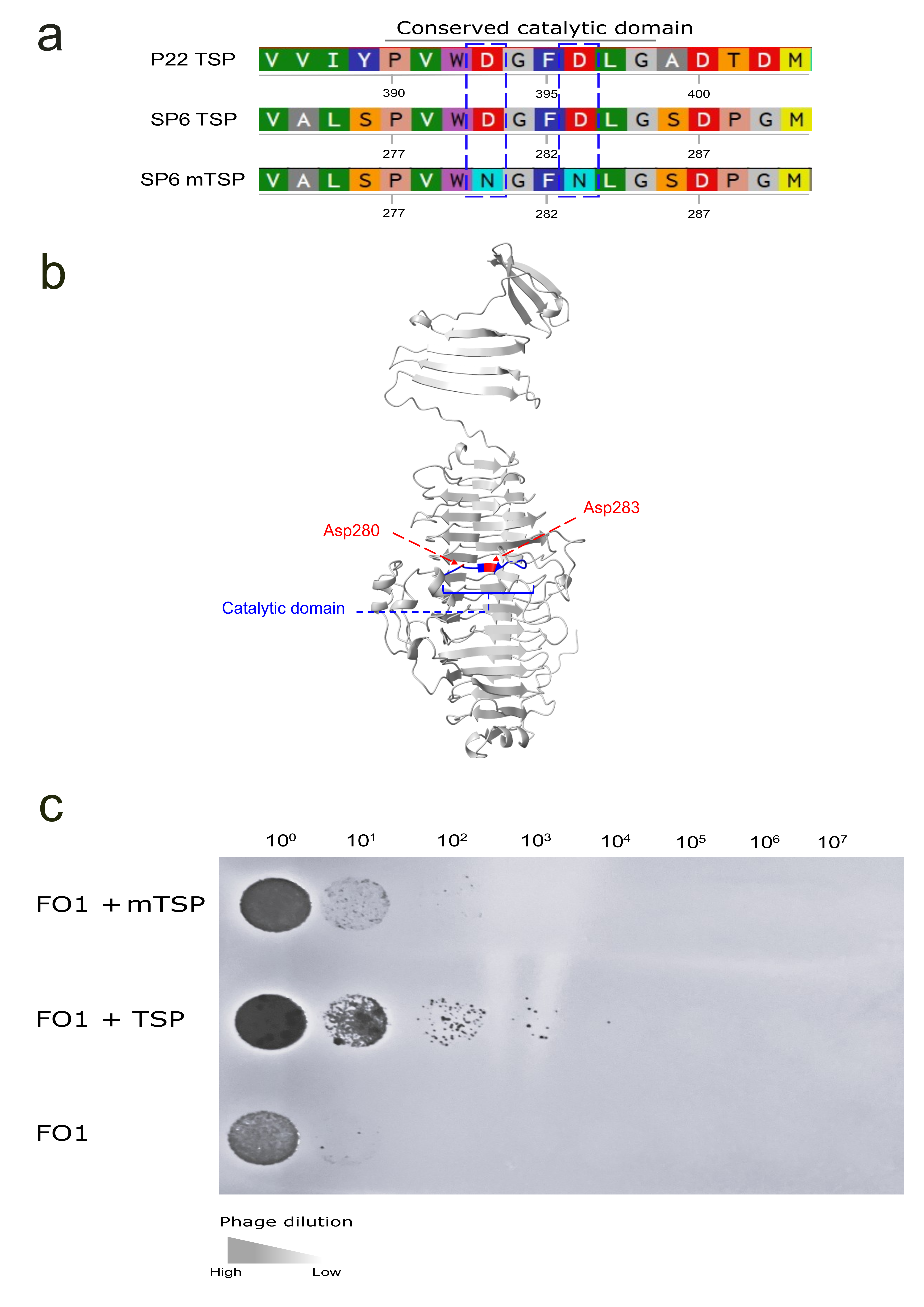

Supplement: Figure S1 — Catalytic domain mutations in SP6 TSP reduce FO1 infectivity. [file aem.01384-25-s0001.tif]

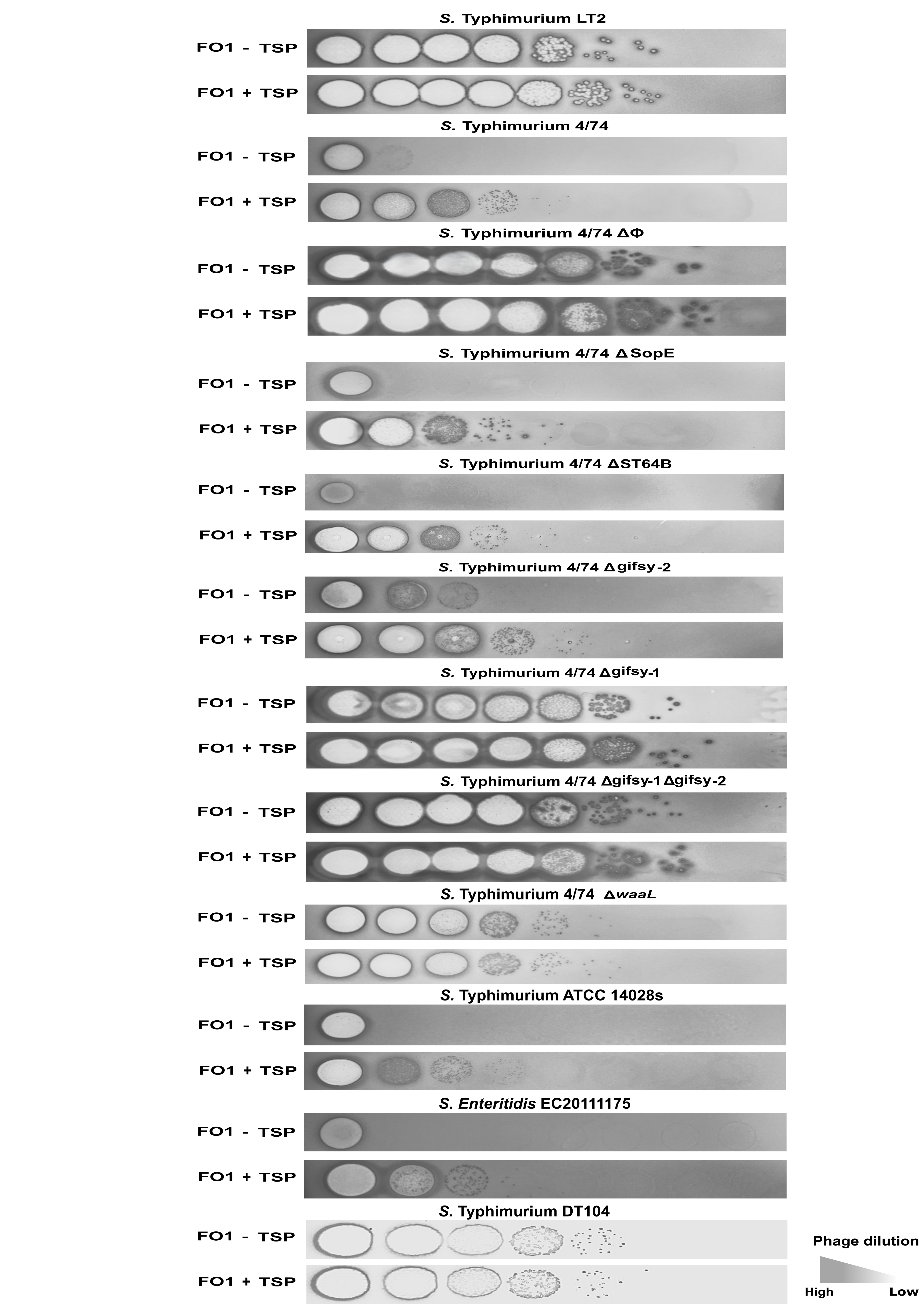

Supplement: Figure S2 — Plaque assay SP6 TSP enhancing infectivity of phage FO1 across diverse Salmonella strains. [file aem.01384-25-s0002.tif]

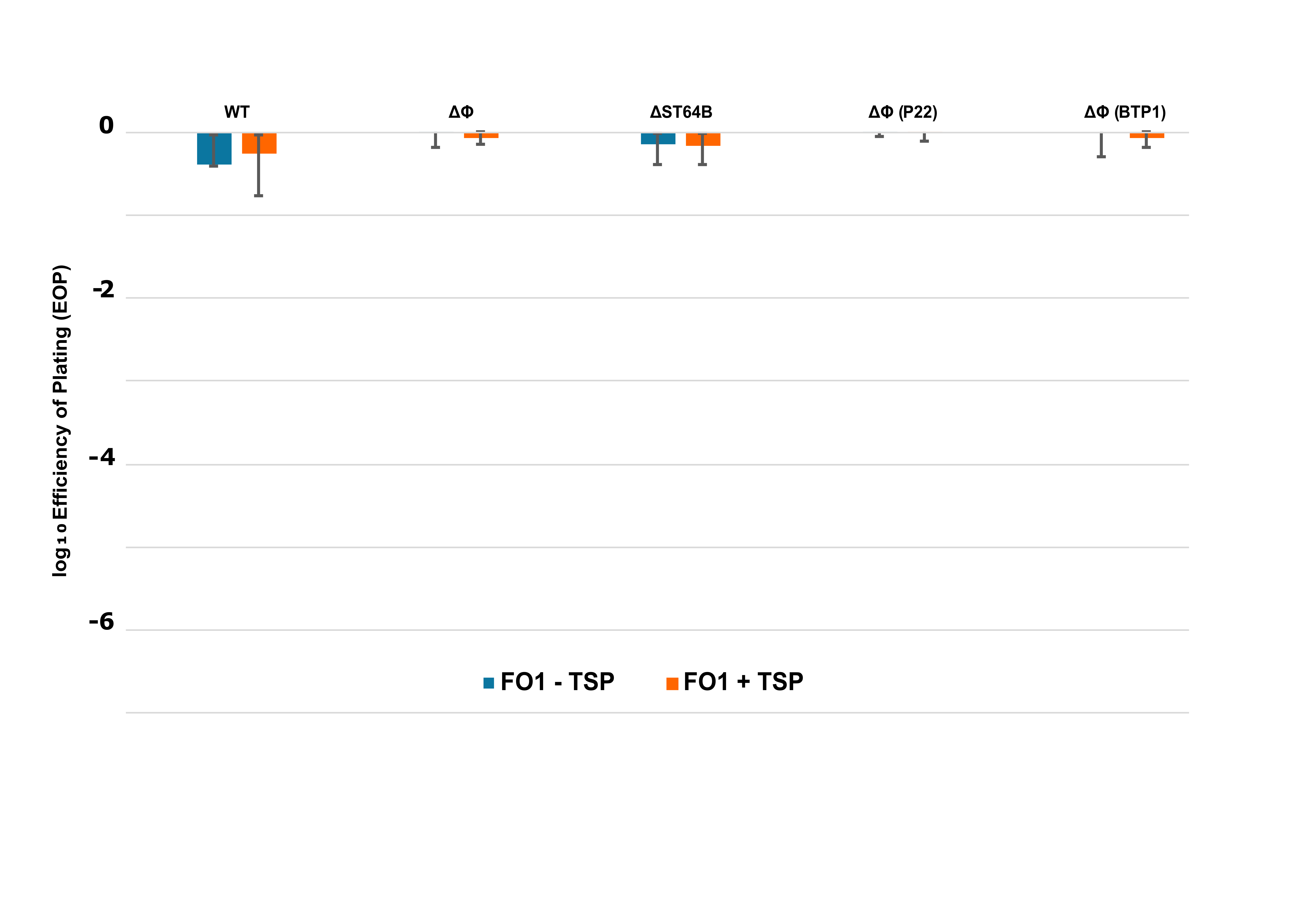

Supplement: Figure S3 — Supplementation of SP6 TSP does not enhance FO1 infectivity on S. Typhimurium D23580 or its prophage deletion mutants. [file aem.01384-25-s0003.tif]

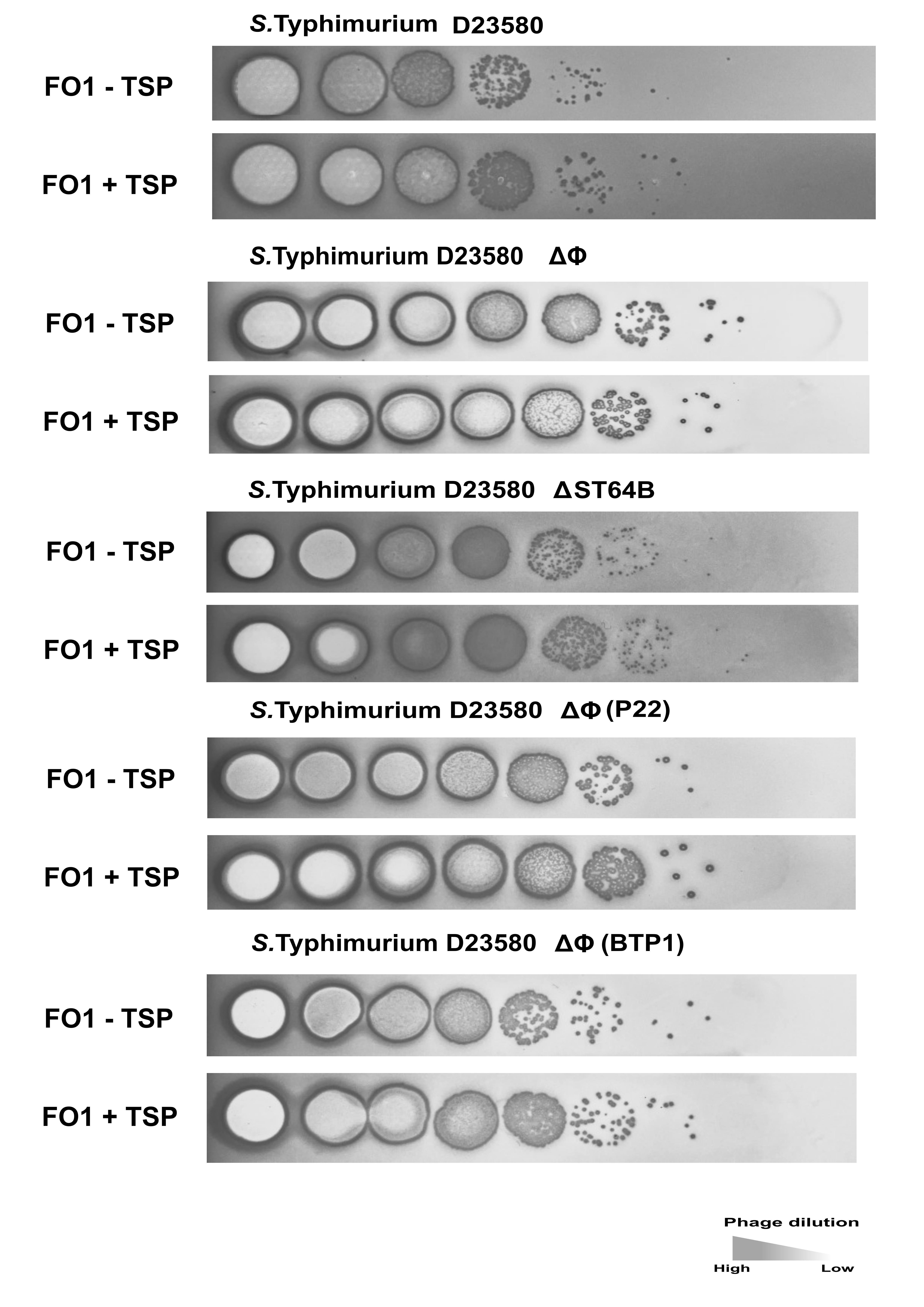

Supplement: Figure S4 — Plaque assay SP6 TSP enhancing infectivity of phage FO1 on S. Typhimurium D23580, its prophage deletion strains, and lysogenized strains. [file aem.01384-25-s0004.tif]

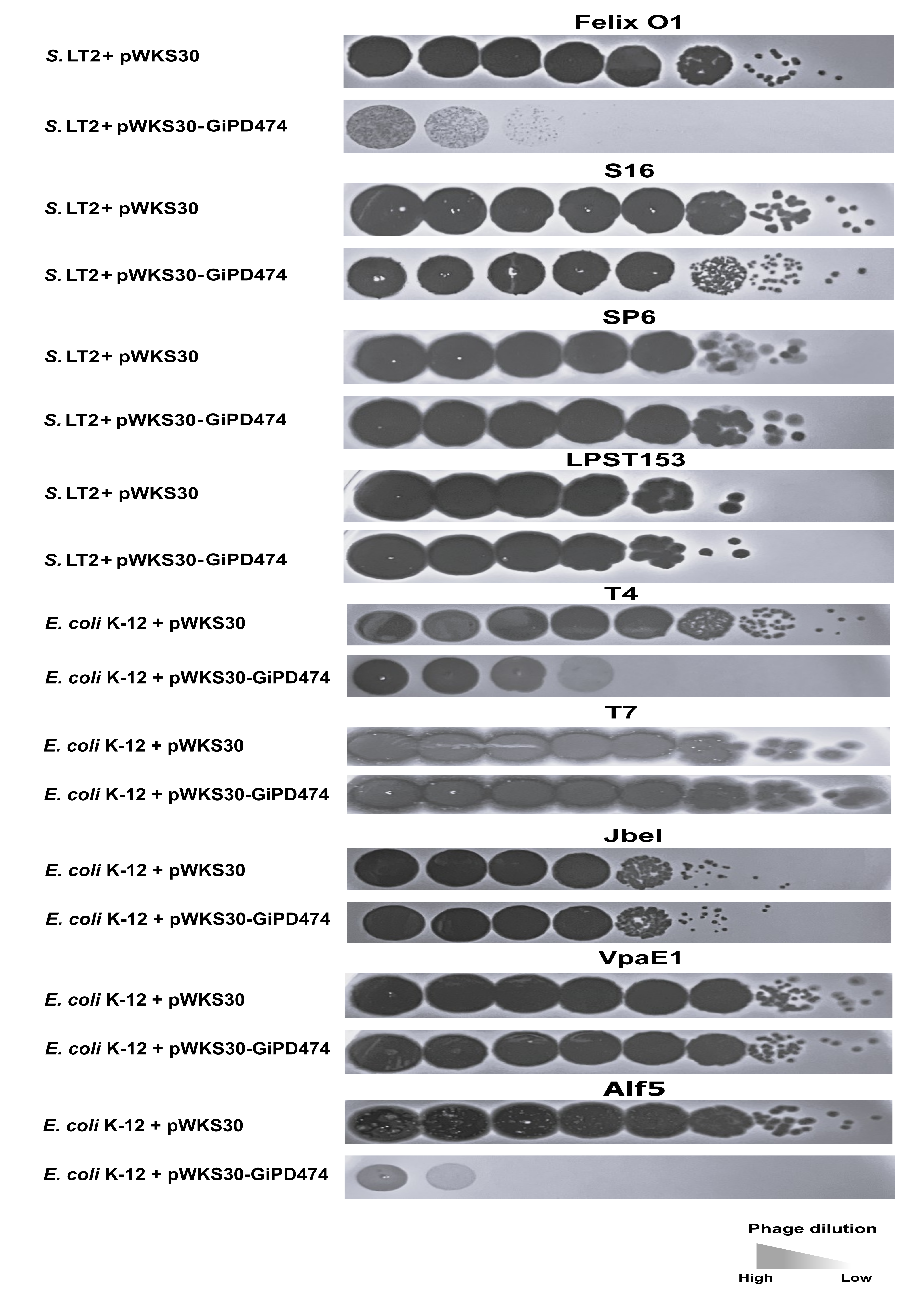

Supplement: Figure S5 — Spot assay demonstrates that GiPD474 confers resistance to diverse lytic bacteriophages onto bacterial hosts S. Typhimurium LT2 and E. coli K-12. [file aem.01384-25-s0005.tif]

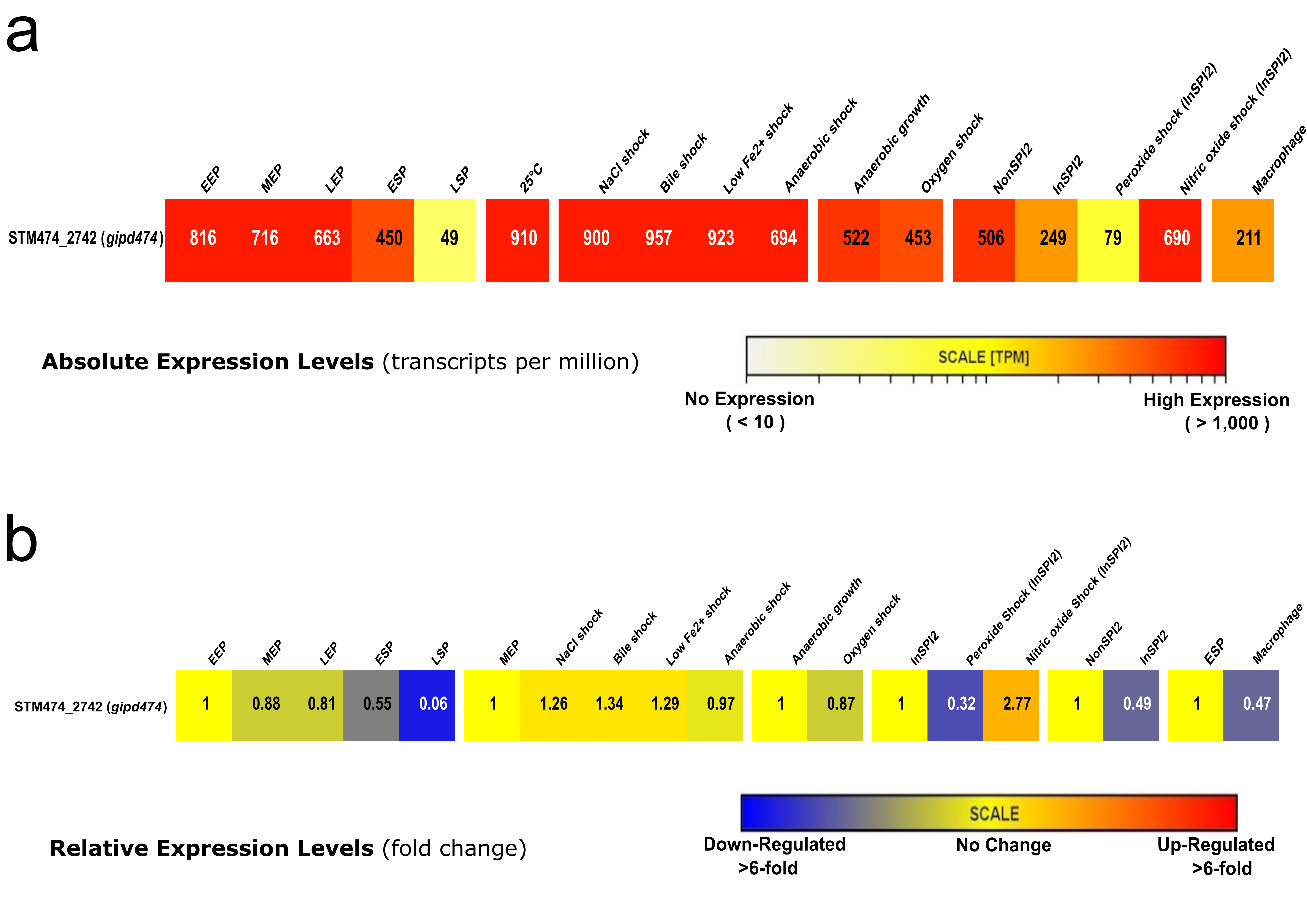

Supplement: Figure S6 — Absolute and relative expression of STM474_2742 (gipd474) in Salmonella Typhimurium 4/74 under various growth and stress conditions obtained from the SalComD23580 transcriptome compendium. [file aem.01384-25-s0006.tif]
