# Supplementary material for: A Gifsy prophage-encoded protein confers broad phage resistance in Salmonella enterica and is widely distributed across Enterobacteriaceae
Source: Appl Environ Microbiol. 2025 Nov 10;91(12):e01384-25. doi: 10.1128/aem.01384-25 (PMC12724215; doi:10.1128/aem.01384-25)
Supplement: Supplemental legends — Legends for Fig. S1 to S6. [file aem.01384-25-s0007.docx]

**Supplemental Fig. 1.** Catalytic domain mutations in SP6 TSP reduce FO1 infectivity

enhancement. **(a)** Amino acid sequence alignment comparing phage P22 TSP, SP6 TSP (Gp46), and a catalytically mutated type mTSP. Conserved residues in the catalytic site are highlighted with introduced mutations D280N and D283N in mTSP boxed in blue. **(b)** Predicted 3D model of SP6 TSP monomer with highlighted catalytic domain (blue) and mutated positions Asp280 and Asp 283 (red) in mTSP. **(c)** Functional assay showing plaque formation of FO1 on *S*. Typhimurium 4/74 in the presence of wild-type TSP, mutant TSP (mTSP) or no TSP added. Phage solutions were spotted in 10-fold serial dilutions, with TSP, or mTSP supplemented to a final concentration of 10 ng/µl per spot.

**Supplemental Fig. 2.** Plaque assay SP6 TSP enhancing infectivity of phage FO1 across diverse

*Salmonella* strains. Purified FO1 phage particles were spotted onto a bacterial lawn of various strains of *S. enterica*, including *S.* Typhimurium and *S.* Enteritidis. Phage solutions were spotted in 10-fold serial dilutions without TSP or with TSP supplemented to a final concentration of 10 ng/µl per spot.

**Supplemental Fig. 3.** Supplementation of SP6 TSP does not enhance FO1 infectivity on S. Typhimurium

D23580 or its prophage deletion mutants. Log_10_ efficiency of plating values calculated for FO1 with and without

SP6 TSP supplementation on *S.* Typhimurium D23580 and prophage deletion mutants, and reference strain *S.* Typhimurium LT2. TSP was supplemented to a final concentration of 10 ng/µl. Bars represent mean EOP values from biological and technical replicates and error bars indicate standard deviation.

**Supplemental Fig. 4** Plaque assay SP6 TSP enhancing infectivity of phage FO1 on *S*. Typhimurium

D23580, its prophage deletion strains, and lysogenized strains. Purified FO1 phage particles were spotted onto the bacterial lawn of *S.* Typhimurium D23580, including prophage deletion strains and lysogenized strains ΔΦ (P22) and ΔΦ (BTP1). Phage solutions were spotted in 10-fold serial dilutions without TSP or with TSP supplemented to a final concentration of 10 ng/µl per spot.

**Supplemental Fig. 5.** Spot assay demonstrates that GiPD474 confers resistance to diverse lytic bacteriophages onto bacterial hosts *S.* Typhimurium LT2 and *E. coli* K-12. Tenfold phage solution was spotted onto bacterial host complemented with empty vector pWKS30 (reference strain) or *gipd474* expression plasmid pWKS30-GiPD474 (test strain). Spot test assays were performed in both biological and technical triplicates.

**Supplemental Fig. 6**. Absolute and relative expression of STM474_2742 (*gipd474*) in *Salmonella*

Typhimurium 4/74 under various growth and stress conditions obtained from the SalComD23580 transcriptome compendium. (**a**): Absolute expression levels shown as transcripts per million (TPM), highlighting strong transcription (>500 TPM) during exponential growth and stress responses such as NaCl, bile, and iron limitation. (**b**) Intra-strain relative fold-change compared to early exponential phase (EEP), indicating SPI2-specific upregulation and repression during late stationary phase (LSP). Growth phases are abbreviated as EEP (early exponential phase), MEP (mid exponential phase), LEP (late exponential phase), ESP (early stationary phase), and LSP (late stationary phase).
