# Supplementary material for: A Gifsy prophage-encoded protein confers broad phage resistance in Salmonella enterica and is widely distributed across Enterobacteriaceae
Source: Appl Environ Microbiol. 2025 Nov 10;91(12):e01384-25. doi: 10.1128/aem.01384-25 (PMC12724215; doi:10.1128/aem.01384-25)
Supplement: Table S1 — Strains and plasmids used in this study. [file aem.01384-25-s0008.docx]

**Table S1 Strains and plasmids used in this study.**

| **Bacterial strains** | **Characteristic** | **Source** | **Reference** |
| --- | --- | --- | --- |
| ***Escherichia coli* strains** |  |  |  |
| DH5ɑ | Cloning strain | New England Biolabs |  |
| str. K-12 substr. MG1655 | Wild type | Lidija Truncaite | (74) |
| BE | Wild type | Lidija Truncaite | (54) |
| ***Salmonella enterica ser.* Typhimurium strains** |  |  |  |
| LT2 strain LB5000 | r⁻ m⁺ for hsdLT, hsdSA, hsdSB | SGSC 181 | (77) |
| 4/74 | Wild type | Jay Hinton | (46) |
| 4/74 ΔΦ | 4/74 mutant prophage deficient | Jay Hinton |  |
| 4/74 ΔSopE | 4/74 mutant prophages SopE deletion | Jay Hinton |  |
| 4/74 ΔGifsy-1 ΔGifsy-2 | 4/74 mutant prophages Gifsy-1 and Gifsy-2 deletion | Jay Hinton |  |
| 4/74 ΔGifsy-1 | 4/74 mutant prophages Gifsy-1 deletion | Jay Hinton |  |
| 4/74 ΔGifsy-2 | 4/74 mutant prophages Gifsy-2 deletion | Jay Hinton |  |
| 4/74 ΔST64B | 4/74 mutant prophage ST64B deletion | This study |  |
| 4/74 Δ*waaL* | 4/74 mutant gene *waaL* deletion | This study |  |
| 4/74 Δ*GiPD474* | 4/74 mutant gene *GiPD474* deletion | This study |  |
| 14028S | Wild type | ATCC 14028 | (64) |
| ST131 str. D23580 | Wild type | Jay Hinton | (47) |
| ST131 str. D23580 ΔΦ | 4/74 mutant prophage deficient | Jay Hinton |  |
| ST131 str. D23580 ΔST64B | 4/74 mutant prophage ST64B deletion | Jay Hinton |  |
| ST131 str. D23580 ΔΦ (P22) | 4/74 mutant prophage deficient P22 lysogen | Jay Hinton |  |
| ST131 str. D23580 ΔΦ (BTP1) | 4/74 mutant prophage deficient BTP1 lysogen | Jay Hinton |  |
| DT104 | Wild type | PDX | (78) |
| ***Salmonella enterica ser. Enteritidis* strain** |  |  |  |
| EC20111175 | Wild type | PDX | (76) |
| **Bacteriophages** |  |  |  |
| Felix O1 (DSM 18524) | Host: *S.* LT2 | DSMZ | (79) |
| SP6 | Host: *S.* LT2 | Félix d'Hérelle Reference Center | (80) |
| S16 | Host: *S.* LT2 | Félix d'Hérelle Reference Center | (81) |
| LPST153 | Host: *S.* LT2 | Jinquan Li | (57) |
| Jbel | Host: *E. coli* K12 | Bowden lab | (56) |
| T4 | Host: *E. coli* K12 | Vincent Noireaux | (51) |
| T7 | Host: *E. coli* K12 | Vincent Noireaux | (82) |
| Alf5 | Host: *E. coli* K12 | Lidija Truncaite | (55) |
| VpaE1 | Host: *E. coli* BE | Lidija Truncaite | (54) |
| ***Plasmids*** |  |  |  |
| pKD4 | *kmR*, *ampR*, ColE1 ori, FRT sites | Datsenko and Wanner | (84) |
| pKD46 | *ampR*, pBAD, pSC101ts, λ Red recombinase (gam, bet, exo), FRT sites | Datsenko and Wanner | (84) |
| pWKS30 | *ampR*, pSC101, *lacZα*, pLac, MCS, | Wang and Kusher | (85) |
| pWKS30-Remain | *ampR*, pSC101, *lacZα*, pLac, remAIN genes (*remA, remI and remN)* | This study |  |
| pWKS30-GiPD474 | *ampR,* pSC101*, lacZα,* pLac*, STM474_RS13505* gene *(gipd474)* | This study |  |
