# Supplementary material for: A Gifsy prophage-encoded protein confers broad phage resistance in Salmonella enterica and is widely distributed across Enterobacteriaceae
Source: Appl Environ Microbiol. 2025 Nov 10;91(12):e01384-25. doi: 10.1128/aem.01384-25 (PMC12724215; doi:10.1128/aem.01384-25)
Supplement: Table S2 — Primers used in this study. [file aem.01384-25-s0009.docx]

**Table S2 Primers used in this study.**

| **Primer name** | **Sequence (5'-3')** | **Usage** | **Notes/Target** |
| --- | --- | --- | --- |
| FP1_gp46_flag1 | AGGGCTGGATAGTAATAGGAGGTTTAATGCACCACCACCACCACCACGGTAGCGGTTCATTAACTAAACCACGTTGCTTCAG | Synthetic DNA construct | *gp46*, His tag |
| FP2_gp46_flag2 | CCTCTGGCGGTGATAATGGTTGCATTCACGGGGAGGGCTGGATAGTAATAGGAGG | Synthetic DNA construct | *gp46*, His tag, UTR, p70a |
| FP3_gp46_flag3 | GGAAAATTTTTTTAAAAAAAGAATCTTGACAATTTTACCTCTGGCGGTGATAATGG | Synthetic DNA construct | *gp46*, His tag, UTR, p70a, UP-element |
| RP1_gp46_flag1 | ACAGAAAAGCCCGCCTTTCGGCGGGCTTTGCTGAAAGGCTACCACTATGATAACC | Synthetic DNA construct | *gp46*, T500 terminator |
| RP1_gp46 | CTGAAAGGCTACCACTATGATAACC | Synthetic DNA construct | *gp46* |
| FP1_gp46 | CATTAACTAAACCACGTTGCTTCAG | Site directed PCR mutagenesis | *gp46* |
| RP1_mutant_gp46 | GCCTAAGTTAAAGCCGTTCCATAC | Site directed PCR mutagenesis | Containing mutations |
| FP1_mutant_gp46 | GTATGGAACGGCTTTAACTTAGGC | Site directed PCR mutagenesis | Containing mutations |
| FP_waaL_KO | ATGCTAACCACATCATTAACGTTAAATAAAGAGAAATGGAGTGTAGGCTGGAGCTGCTTC | λ Red recombination | *waaL* disruption |
| RP_waaL_KO | TCTATTTCTTAGCGCCAGCAGAAAACCGGTAATGATACATGGGAATTAGCCATGGTCC | λ Red recombination | *waaL* disruption |
| FP_ST64B_KO | GTTAGCGCGGTAATCCCGTTTTTTAACTCCCTTCCGGTTAGTGTAGGCTGGAGCTGCTTC | λ Red recombination | ST64B disruption |
| RP_ST64B_KO | ACGCTTCCTGTCCAGGGTTGGGCTCATTTTCACCTTGCGACATGGGAATTAGCCATGGTCC | λ Red recombination | ST64B disruption |
| FP_GiPD474_KO | GATAATGGACGTGTTGAAGCTACTGATAGGCCAACAGAAAGTGTAGGCTGGAGCTGCTTC | λ Red recombination | *gipd* disruption |
| RP_GiPD474_KO | AAACATTTAACAACTGTAGATACGTCAGATTCTAGCGCTCATGGGAATTAGCCATGGTCC | λ Red recombination | *gipd474* disruption |
| P1_waaL_KO | CATGCTGCTCACCAGAACAG | λ Red recombination | Verify disruption of *waaL* |
| P1_ST64_KO | CGTCACTTGAACAAAATTACCCG | λ Red recombination | Verify disruption of ST64B |
| P1_ GiPD474_KO | GGTTTTTGTGTCCACCAGTG | λ Red recombination | Verify disruption of *gipd474* |
| FP2_KanR | CTACCCGTGATATTGCTGAAGAG | λ Red recombination | Target KmR insert |
| RP2_KanR | CTCTTCAGCAATATCACGGGTAG | λ Red recombination | Target KmR insert |
| FP_pWKS30_BsmBIrem | GGCTACCGTCTCATGCTATGTTGAGCCAGCTTTTGTTCCCTTTAG | Golden Gate Cloning | pWKS30 + BsmBI sites |
| RP_pWKS30_BsmBIrem | GGCTACCGTCTCACAATTCGCCCTATAGTGAG | Golden Gate Cloning | pWKS30 + BsmBI sites |
| FP_remAIN_BsmBI | GGCTACCGTCTCAATTGGGTTAAATTAGAGGTGTTAGTTC | Golden Gate Cloning | remAIN + BsmBI sites |
| RP_remAIN_BsmBI | GGCTACCGTCTCAAGCAAGGAATAATTATGCC | Golden Gate Cloning | remAIN + BsmBI sites |
| FP_pWKS30_BsmBIpip | GGCTACCGTCTCATTGTTCCCTTTAGTGAGG | Golden Gate Cloning | pWKS30 + BsmBI sites |
| RP_pWKS30_BsmBIpip | GGCTACCGTCTCATTCGCCCTATAGTGAGTC | Golden Gate Cloning | pWKS30 + BsmBI sites |
| FP_ GiPD474_BsmBI | GGCTACCGTCTCACGAATTGGGCCGTGTAAGTAAAGCCG | Golden Gate Cloning | *gipd474 +* BsmBI sites |
| RP_ GiPD474_BsmBI | GGCTACCGTCTCAACAAAAGCTGGACAAATACACACAGGATACG | Golden Gate Cloning | *gipd474* + BsmBI sites |
| RP_remAIN_check | ACAACCTCCGTGACCAATATG | Golden Gate Cloning | remAIN insert check |
| RP_ GiPD474_check | CATTGGCTCATCACCAGTTAAAG | Golden Gate Cloning | *gipd474* gene insert check |
| FP_pWKS30_bb | CAAGGAAAGAACGGACGGTATC | Golden Gate Cloning | Target pWKS30 backbone |

* Underlined sequences indicate homology arms for λ Red recombination
